# Identification of a cancer-associated fibroblast classifier for predicting prognosis and therapeutic response in lung squamous cell carcinoma

**DOI:** 10.1097/MD.0000000000035005

**Published:** 2023-09-22

**Authors:** Xixi Lai, Gangze Fu, Haiyan Du, Zuoliu Xie, Saifeng Lin, Qiao Li, Kuailu Lin

**Affiliations:** a Department of Respiratory and Critical Medicine, The First Affiliated Hospital of Wenzhou Medical University, Wenzhou, China; b Department of Visceral, Thoracic and Vascular Surgery, Carl Gustav Carus University Hospital Dresden, Technische Universität Dresden, Dresden, Germany; c Department of Radiology, The First Affiliated Hospital of Wenzhou Medical University, Wenzhou, China; d Department of Ultrasonography, The First Affiliated Hospital of Wenzhou Medical University, Wenzhou, China; e Department of Breast Surgery, The First Affiliated Hospital of Wenzhou Medical University, Wenzhou, China.

**Keywords:** cancer-associated fibroblasts, lung squamous cell carcinoma, PI3K-Akt signaling, prognosis, weighted gene co-expression network analysis

## Abstract

Reliable prognostic gene signatures for cancer-associated fibroblasts (CAFs) in lung squamous cell carcinoma (LUSC) are still lacking, and the underlying genetic principles remain unclear. Therefore, the 2 main aims of our study were to establish a reliable CAFs prognostic gene signature that can be used to stratify patients with LUSC and to identify promising potential targets for more effective and individualized therapies. Clinical information and mRNA expression were accessed of the cancer genome atlas-LUSC cohort (n = 501) and GSE157011 cohort (n = 484). CAFs abundance were quantified by the multi-estimated algorithms. Stromal CAF-related genes were identified by weighted gene co-expression network analysis. The least absolute shrinkage and selection operator Cox regression method was utilized to identify the most relevant CAFs candidates for predicting prognosis. Chemotherapy sensitivity scores were calculated using the “pRRophetic” package in R software, and the tumor immune dysfunction and exclusion algorithm was employed to evaluate immunotherapy response. Gene set enrichment analysis and the Search Tool for Interaction of Chemicals database were applied to clarify the molecular mechanisms. In this study, we identified 288 hub CAF-related candidate genes by weighted gene co-expression network analysis. Next, 34 potential prognostic CAFs candidate genes were identified by univariate Cox regression in the cancer genome atlas-LUSC cohort. We prioritized the top 8 CAFs prognostic genes (DCBLD1, SLC24A3, ILK, SMAD7, SERPINE1, SNX9, PDGFA, and KLF10) by a least absolute shrinkage and selection operator Cox regression model, and these genes were used to identify low- and high-risk subgroups for unfavorable survival. In silico drug screening identified 6 effective compounds for high-risk CAFs-related LUSC: TAK-715, GW 441756, OSU-03012, MP470, FH535, and KIN001-266. Additionally, search tool for interaction of chemicals database highlighted PI3K-Akt signaling as a potential target pathway for high-risk CAFs-related LUSC. Overall, our findings provide a molecular classifier for high-risk CAFs-related LUSC and suggest that treatment with PI3K-Akt signaling inhibitors could benefit these patients.

## 1. Introduction

Lung cancer is one of the most prevalent malignancies worldwide. Approximately 2.1 million new cases and 1.8 million cancer-related deaths are reported annually, accounting for 18.4% of all cancer deaths.^[1]^ Lung squamous cell carcinoma (LUSC) is the most common type of lung cancer, representing approximately 40% of all lung cancers. LUSC is associated with a poor clinical outcome, particularly in the absence of targeted agents, compared with lung adenocarcinoma.^[2,3]^ Since the early 2000s, the development of molecular inhibitors targeting epidermal growth factor receptor, vascular endothelial growth factor, and anaplastic lymphoma kinase has significantly improved the prognosis of patients with lung adenocarcinoma. However, progress in the treatment of LUSC has remained limited.^[4–6]^ Immunotherapy is a promising strategy for cancer treatment and has recently been used for LUSC.^[7]^ Immune checkpoint inhibitors, such as agents targeting cytotoxic T-lymphocyte associated protein 4, programmed cell death-ligand-1, and programmed cell death-1, have demonstrated impressive improvements in survival outcomes for patients with advanced LUSC.^[8]^ These molecules play crucial roles in immune evasion mechanisms utilized by cancer cells and, by inhibiting these molecules, immune checkpoint inhibitors can effectively activate the patient’s immune system to recognize and eliminate tumor cells.^[9]^ Ongoing research in this area holds great promise for advancing our understanding of the underlying mechanisms that drive LUSC development and progression, with the ultimate goal of developing more effective treatment strategies for patients afflicted with this disease.^[10]^

Cancer-associated fibroblasts (CAFs) constitute a significant portion of the tumor stroma in LUSC and play a critical role in the development and progression of this disease by modulating the tumor microenvironment (TME).^[11,12]^ CAFs exhibit a diverse range of functions, including recruitment of immunosuppressive cells (such as myeloid-derived suppressor cells^[13]^and regulatory T cells^[14]^) and inhibition of the activity of effector immune cells (such as cytotoxic T cells and natural killer cells).^[15,16]^ Additionally, CAFs secrete various cytokines and growth factors that enhance tumor cell proliferation, angiogenesis, and invasion.^[15]^ Because of their multifaceted role in tumor progression, CAFs are considered a crucial therapeutic target for improving immune-mediated tumor control in LUSC. Hence, further research on CAFs and their interactions with the TME may yield new insights for the development of more effective treatment strategies for LUSC.^[17]^ Silicon analysis has been successfully applied to identify CAF gene markers and stromal infiltrations in some tumor entities^[18–20]^; however, reliable prognostic gene signatures for CAFs in LUSC are currently lacking, and the underlying genetic principles that govern their behavior in this context remain unclear.

Therefore, the 2 main aims of our study were to establish a reliable CAF prognostic gene signature that can be used to stratify patients with LUSC and to identify promising potential targets for more effective and individualized therapies. We identified 34 survival-related CAF gene expression profiles and used a least absolute shrinkage and selection operator (LASSO) Cox regression model to prioritize 8 candidate genes, ultimately establishing a robust risk model for LUSC. Finally, we conducted somatic mutation and copy number variation (CNV) analysis and proposed potential sensitive chemotherapy and immunotherapy agents for individual LUSC CAF risk clusters. Through these efforts, we aimed to pave the way for more precise and targeted treatment approaches for patients with LUSC, ultimately improving outcomes.

## 2. Methods

### 2.1. Acquisition and preprocessing of clinical and expression datasets

Clinical information and mRNA expression were accessed from https://portal.gdc.cancer.gov/ for the cancer genome atlas (TCGA)-LUSC cohort (n = 501) in January 2022. Normalized transcriptome and clinical data of 484 patients with LUSC in the GSE157011 cohort were obtained from the Gene Expression Omnibus database in January 2022.^[21]^

### 2.2. Collection of CAF markers and calculation of CAF abundance

#### 2.2.1. Collection of CAF markers.

In total, 59 CAF-related nonspecific and specific candidate markers were collected from previously published reports.^[22,23]^ These markers were utilized to compute CAF infiltrations using 2 distinct algorithms: the microenvironment cell populations-counter (MCP-counter), and single-sample gene set enrichment analysis (ssGSEA).

#### 2.2.2. Estimating the proportions of immune and cancer cells (EPIC) method.

EPIC CAF infiltration scores for the TCGA-LUSC and GSE157011 cohorts were scored by the “EPIC” package in R software.^[24]^

#### 2.2.3. MCP-counter.

MCP-counter CAF abundance scores for the TCGA-LUSC and GSE157011 cohorts were scored by the “MCPcounter” package in R software.^[25]^

#### 2.2.4. Tumor immune dysfunction and exclusion (TIDE) computational framework.

TIDE CAF abundance levels for the TCGA-LUSC and GSE157011 cohorts were estimated through http://tide.dfci.harvard.edu/.

#### 2.2.5. ssGSEA.

Enrichment scores for individual cases were computed by ssGSEA through application of the “GSVA” package in R software,^[26]^ and published CAF markers were used for calculation.

#### 2.2.6. Stromal scores.

The stromal scores of the TCGA-LUSC and GSE157011 cohorts were computed by the “estimate” package in R software.^[27]^

#### 2.2.7. Weighted gene co-expression network analysis (WGCNA) networks.

Co-expression networks targeting multi-estimated CAF abundance levels and stromal scores were identified and constructed by the “WGCNA” package in R software.^[28]^ Initially, genes with the top 10,000 median absolute deviations were collected as input genes for the TCGA-LUSC and GSE157011 cohorts individually, and “mergeCutHeight” was set at 0.5.

### 2.3. Survival analyses

The optimal cutoffs of the LASSO risk score for overall survival (OS) in the TCGA-LUSC and GSE157011 cohorts were determined using “maxstat” with abseps = 0.01.

Kaplan–Meier curves were plotted, and a log-rank test was used to compute differences in OS between the groups. This was performed by the “survival,” “survminer,” and “ggplot2” packages in R software.

Univariate Cox regression analysis and hazard ratios with 95% confidence intervals were calculated using the “survival” package in R software.

### 2.4. Single-cell sequencing analysis

The tumor immune single-cell hub 2 online database (http://tisch.comp-genomics.org/) ^[29]^was utilized to analyze the single-cell sequencing data from 5 patients with LUSC (EMTAB6149).

### 2.5. Differential expression analysis

Differentially expressed mRNAs were calculated by the “EdgeR” package in R software.^[30]^

### 2.6. LASSO Cox regression

The LASSO Cox regression method was utilized to identify the most relevant CAF candidates for predicting prognosis using the “glmnet” package in R software.^[31,32]^

The risk score was calculated by the “glmnet” package in R software (s = lambda. min and type = “response”). The analytical formula for risk assessment was based on 8 prioritized candidates (coefficients of genes: DCBLD1 = 0.104627493363669, SLC24A3 = 0.00870174771643035, ILK = 0.0409692300913776, SMAD7 = 0.0424186193770709, SERPINE1 = 0.0273947439430555, SNX9 = 0.0903772085761147, PDGFA = 0.0083020522296611, and KLF10 = 0.018161946159685).

### 2.7. Somatic mutation and CNV analysis

The top 200 high-frequency somatic mutations in the TCGA-LUSC cohort were accessed from cBioPortal in January 2022. Significant enrichment among distinct CAF prognostic groups was analyzed by the chi-square test.

Global CNV data of the TCGA-LUSC cohort were accessed from http://www.firebrowse.org/ in January 2022. A segment mean of > 0.2 was defined as amplification, and a segment mean of < −0.2 was defined as deletion.

Distinct prognostic clusters were identified using Fisher exact test and analyzed by CoNVaQ (https://convaq.compbio.sdu.dk/).^[33]^

CNV plots were visualized using Integrative Genomics Viewer 2.16.0.^[34]^

### 2.8. Gene set enrichment analysis (GSEA)

A GSEA algorithm was performed to determine the normalized enrichment score and statistical significance in the molecular signatures database C5 collection (ontology gene set), Hallmark gene set, and Kyoto encyclopedia of genes and genomes (KEGG) collection terms through the “clusterProfiler” package of R software.^[35]^

### 2.9. Prediction of chemotherapy and immunotherapy sensitivity

Chemotherapy sensitivity scores were computed by the “pRRophetic” package in R software.^[36]^ Immunotherapy predictions in the TCGA-LUSC and GSE157011 cohorts were performed by TIDE through http://tide.dfci.harvard.edu/.

### 2.10. The search tool for interaction of chemicals (STITCH) analysis

Analysis of the interaction networks of chemical compounds and proteins^[37]^ was performed by STITCH (http://stitch.embl.de/).

## 3. Results

### 3.1. Establishment of CAF-related gene signature based on WGCNA

**Figure 1.**
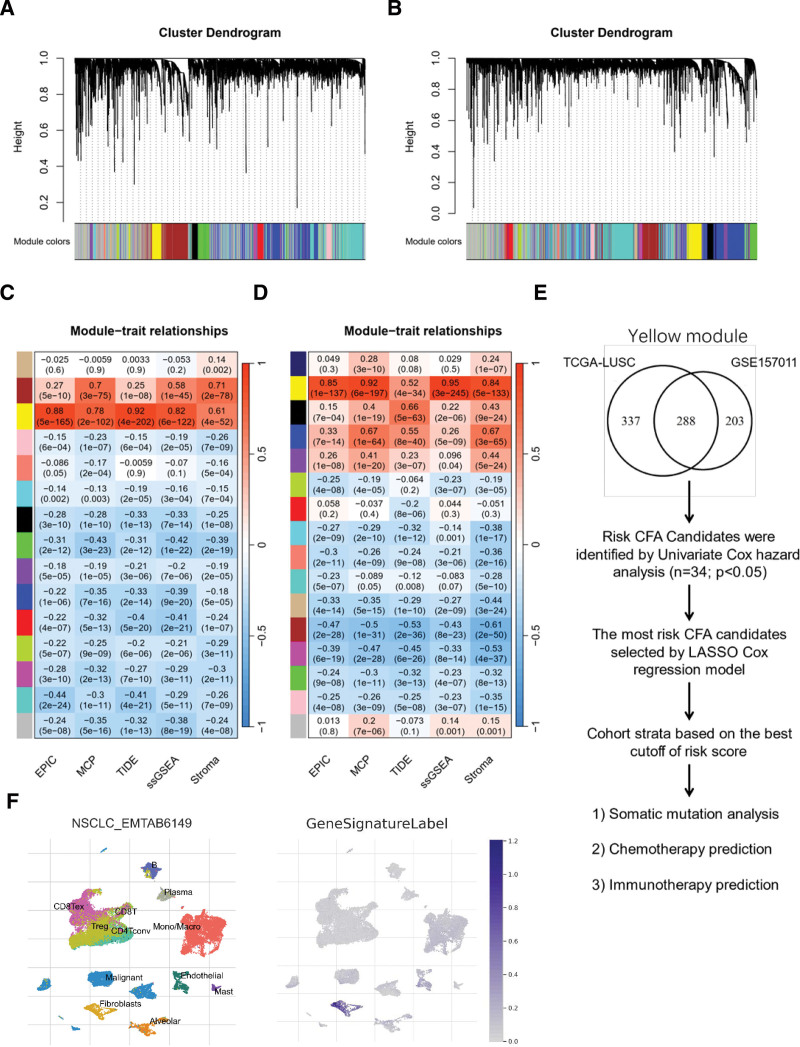
Establishment of CFAs-related candidate genes based on WGCNA. Clustering dendrograms presenting candidates with close expression patterns were clustered into WGCNA modules in TCGA-LUSC (A) and GSE157011 (B) in which gray module genes indicate not assigned in other modules. The correlations between each module eigengenes and phenotypes were presented by heatmaps of module-trait relationships for TCGA-LUSC (C) and GSE157011 (D). Schematic summary of the workflow to compute CFAs-related genes, 288 candidates intersected in yellow module between TCGA-LUSC and GSE157011 (E). Uniform manifold approximation and projection (UMAP) plot of cells from five NSCLC patients (EMTAB6149) reveals consistent clusters of stromal, immune and tumor cells across samples. The CFA-related gene signature mainly labeled in fibroblasts (F). LUSC = lung squamous cell carcinoma, TCGA = the cancer genome atlas, WGCNA = weighted gene co-expression network analysis, NSCLC = non-small cell lung cancer.

WGCNA was performed in the TCGA-LUSC and GSE157011 cohorts. Genes with the top 10,000 median absolute deviations were selected as the candidates for WGCNA network constructions in both cohorts. Five obvious outlier samples revealed by a hierarchical clustering tree were eliminated in the TCGA-LUSC cohort; no samples were eliminated in the GSE157011 cohort. To construct a scale-free topology network, the soft thresholding power (β) of 5 in the TCGA-LUSC cohort (scale-free R^2^ = 0.97) (Figure S1A, Supplemental Digital Content, http://links.lww.com/MD/J720) and 6 in the GSE157011 cohort (scale-free R^2^ = 0.93) (Figure S2A, Supplemental Digital Content, http://links.lww.com/MD/J721) was estimated. Using a dynamic hybrid cutting approach, 15 co-expression models were clustered in the selected TCGA-LUSC cohort (Fig. 1A, Figure S3A, Supplemental Digital Content, http://links.lww.com/MD/J722, Table S1, Supplemental Digital Content, http://links.lww.com/MD/J723). Notably, the yellow module exhibited the strongest positive correlation with the CAF infiltrations and stromal score (Fig. 1C, Figure S1B, Supplemental Digital Content, http://links.lww.com/MD/J720). Again, using a dynamic hybrid cutting approach, 15 co-expression models were clustered in the GSE157011 cohort (Fig. 1B, Figure S3B, Supplemental Digital Content, http://links.lww.com/MD/J722, Table S2, Supplemental Digital Content, http://links.lww.com/MD/J724), and the yellow module again exhibited the strongest positive correlation with the CAF infiltrations and stromal score (Fig. 1D, Figure S2B, Supplemental Digital Content, http://links.lww.com/MD/J721). In total, 625 and 491 candidates were clustered in the yellow modules of the TCGA-LUSC and GSE157011 cohorts, respectively, 288 of which overlapped in both yellow modules. Next, 288 hub candidates that were highly correlated with the multi-estimated CAF abundance and stromal scores were screened out (Fig. 1E). Analysis of the single-cell sequencing data (EMTAB6149) indicated prominent expression of 288 gene signatures in fibroblasts (Fig. 1F). Gene Ontology enrichment analysis considering the domains of biological process, cellular component, and molecular function revealed extracellular matrix organization, collagen-containing extracellular matrix, and extracellular matrix structural constituent as the top features individually (Figure S4, Supplemental Digital Content, http://links.lww.com/MD/J725, Table S3, Supplemental Digital Content, http://links.lww.com/MD/J726).

### 3.2. Construction of LASSO Cox CAF-related prognostic risk model

**Figure 2.**
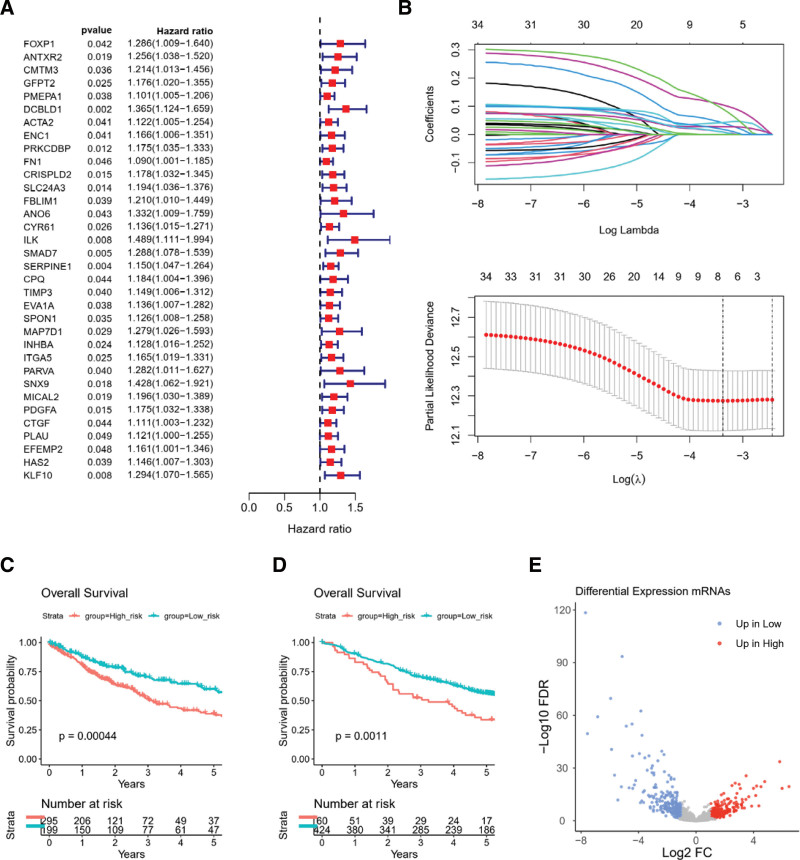
Survival-related CFAs signature established by LASSO Cox model. Forest plot summarizes 34 overall survival-associated CFAs candidates selected by Univariate Cox analysis in TCGA-LUSC (A). The LASSO Cox model revealed 8 most relevant CFAs candidates for OS of TCGA-LUSC cohort (B). Kaplan–Meier plots for five-year OS of the low-risk and high-risk subgroups for TCGA-LUSC (C) and GSE157011 (D), whose strata by the best cutoff algorithm. P-values were computed by the log-rank test. (F) Volcano plot shows significant DEGs (−1 > log2FC > 1, FDR < 0.05, n = 437) among high-risk and low-risk subgroups of TCGA-LUSC computed by edgeR. LASSO = least absolute shrinkage and selection operator, LUSC = lung squamous cell carcinoma, OS = overall survival, TCGA = the cancer genome atlas.

Among the 288 hub candidates, 34 genes were significant with respect to OS in the selected TCGA-LUSC cohort based on univariate Cox analysis: DCBLD1, SERPINE1, SMAD7, ILK, KLF10, PRKCDBP, SLC24A3, PDGFA, CRISPLD2, SNX9, MICAL2, ANTXR2, INHBA, ITGA5, GFPT2, CYR61, MAP7D1, SPON1, CMTM3, EVA1A, PMEPA1, HAS2, FBLIM1, TIMP3, PARVA, ENC1, ACTA2, FOXP1, ANO6, CTGF, CPQ, FN1, EFEMP2, and PLAU (Fig. 2A).

In the selected TCGA-LUSC cohort, we prioritized the most relevant prognostic CAF candidate genes with respect to OS using a LASSO Cox regression model (Fig. 2B). Eight prognostic CAF candidate genes were identified, and all were associated with unfavorable OS: DCBLD1, SLC24A3, ILK, SMAD7, SERPINE1, SNX9, PDGFA, and KLF10.

High-risk and low-risk groups were stratified according to the best cutoff of the LASSO Cox risk score by log-rank analysis in the selected TCGA-LUSC cohort (Fig. 2C) and GSE157011 cohort (Fig. 2D).

### 3.3. Differential gene expression analysis between LASSO CAF risk clusters

In the selected TCGA-LUSC cohort, a differentially expressed gene analysis was performed between the low- and high-risk groups. In total, 437 differentially expressed genes were identified (|log2FC|>=1 and false discovery rate <=0.05), with 249 genes exhibiting higher expression in the low-risk group and 188 genes exhibiting higher expression in the high-risk group (Fig. 2E, Table S4, Supplemental Digital Content, http://links.lww.com/MD/J727).

**Figure 3.**
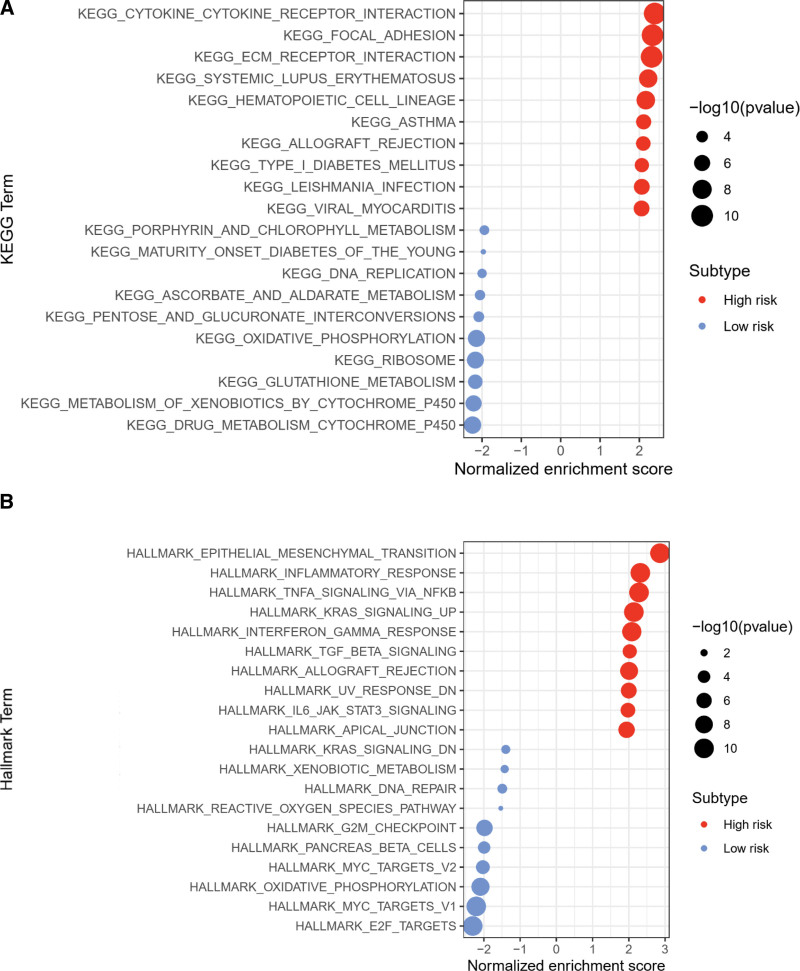
GSEA analysis for DEGs between low-risk or high-risk clusters in TCGA-LUSC cohort. The dot plots present the significant KEGG (A) as well as Hallmark (B) items identified by GSEA algorithm between low-risk or high-risk clusters in TCGA-LUSC cohort. KEGG = Kyoto encyclopedia of genes and genomes, LUSC = lung squamous cell carcinoma, TCGA = the cancer genome atlas.

Gene set enrichment analysis considering the molecular signatures database KEGG and Hallmark datasets revealed that the top terms for the high-risk group were KEGG_CYTOKINE_CYTOKINE_RECEPTOR_INTERACTION and HALLMARK_EPITHELIAL_MESENCHYMAL_TRANSITION, whereas the top terms for the low-risk group were KEGG_DRUG_METABOLISM_CYTOCHROME_P450 and HALLMARK_E2F_TARGETS (Fig. 3A and B; Figure S5A and 5B, Supplemental Digital Content, http://links.lww.com/MD/J728; Table S5, Supplemental Digital Content, http://links.lww.com/MD/J729 and Table S6, Supplemental Digital Content, http://links.lww.com/MD/J730).

### 3.4. High correlation between CAF risk score and CAF infiltration

**Figure 4.**
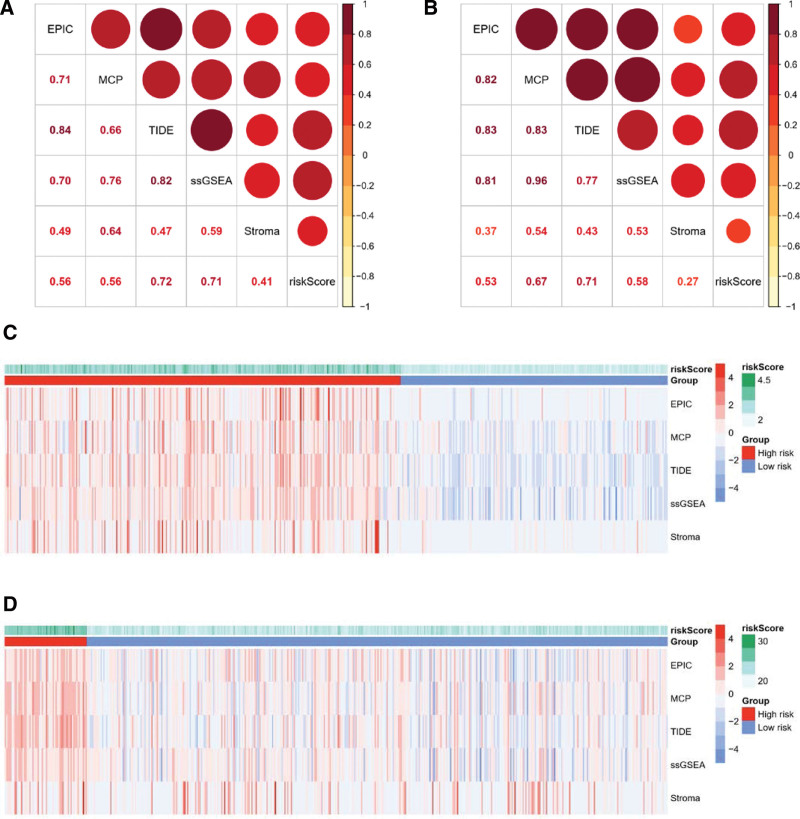
The association between risk stratification as well as risk score and CFAs signatures. Graphs show the LASSO risk score was highly positive correlated with multi-estimated CAFs signatures as well as stromal score in TCGA-LUSC (A) and GSE157011 (B) cohort evidenced by Spearman analysis. Heatmap demonstrates expression pattern of CAFs signatures as well as stromal score stratified into low-risk or high-risk groups in TCGA-LUSC (C) and GSE157011 (D). CAFs = Cancer-associated fibroblasts, LASSO = least absolute shrinkage and selection operator, LUSC = lung squamous cell carcinoma, TCGA = the cancer genome atlas.

To validate the robustness of the CAF risk score for prediction of CAF infiltration, Spearman correlation analyses were conducted between the CAF risk score and multi-estimated CAF infiltrations as well as the stromal score. The risk score was consistently found to have a highly positive correlation with multi-estimated CAF abundances and the stromal score not only in the selected TCGA-LUSC cohort (Fig. 4A) but also in the GSE157011 cohort (Fig. 4B). Moreover, markedly higher multi-estimated CAF abundance levels and stromal scores were found in the high- compared to the low-risk group in both the TCGA-LUSC cohort (Fig. 4C) and GSE157011 cohort (Fig. 4D).

### 3.5. Somatic mutation and CNV analysis between CAF risk clusters

**Figure 5.**
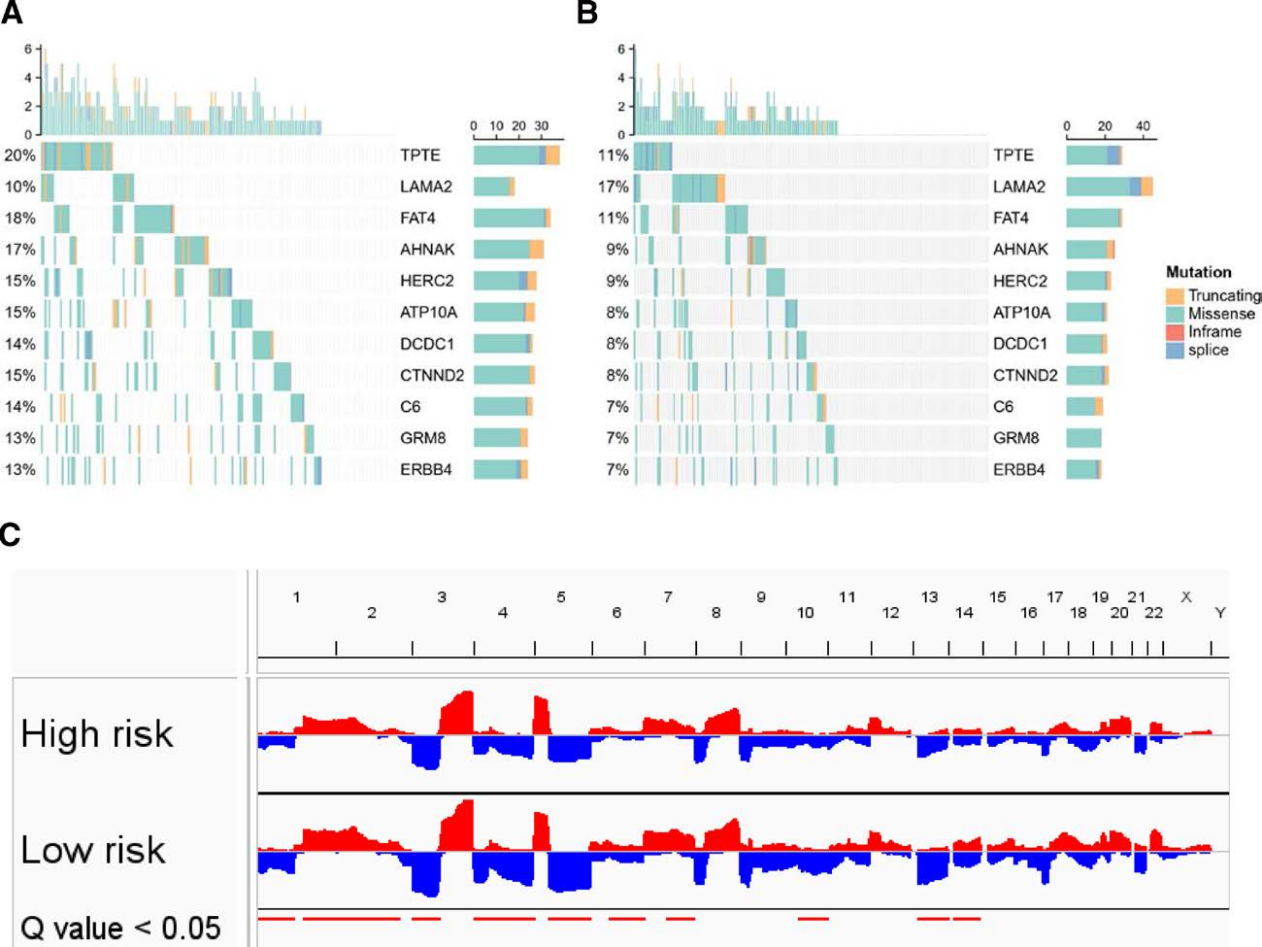
Differences in the mutational landscape among TCGA-LUSC with a high or low-risk score. Oncomaps depicting the significant SNVs of low-risk (A) and high-risk (B) TCGA-LUSCs in top 200 frequency mutational genes. (C) CNA plot shows the relative frequency of copy number gains (red) or deletions (blue) among high-risk and low-risk subgroups of TCGA-LUSC cohort, and displays highly significant differences by Fisher exact test. LUSC = lung squamous cell carcinoma, TCGA = the cancer genome atlas.

To determine whether distinct CAF prognostic phenotypes are the consequence of a differential mutational landscape, we performed an integrative analysis of somatic mutations and CNV. The top 200 high-frequency somatic mutations were analyzed between the high-risk and low-risk groups. Eleven somatic mutations were found to have statistically significant differences between the 2 groups (Fig. 4A and B). Of these 11 mutations, 10 showed significantly higher frequency in the high-risk group (TPTE, FAT4, AHNAK, HERC2, ATP10A, DCDC1, CTNND2, C6, GRM8, and ERBB4), whereas 1 showed higher frequency in the low-risk group (LAMA2). In terms of CNV, several hot spot regions with significant copy number amplifications (chromosomes 1q, 2p, 2q, and 7q) or deletions (chromosomes 1q, 3p, 4p, 4q, 5q, 6q, 10q, 13q, and 14q) were observed as characteristic features of the low-risk group in the TCGA-LUSC cohort (Fig. 5C). Two prognostic CAF-related genes (DCBLD1 and SNX9) in the LASSO Cox regression model located in 6q showed significantly more deletions in the low-risk group (Figure S6A and 6B, Supplemental Digital Content, http://links.lww.com/MD/J732).

### 3.6. Sensitivity of chemotherapy and immunotherapy between CAF risk clusters

We next examined the prediction of resistance or sensitivity of well-established chemotherapy and immunotherapy strategies in the high- and low-risk groups.

**Figure 6.**
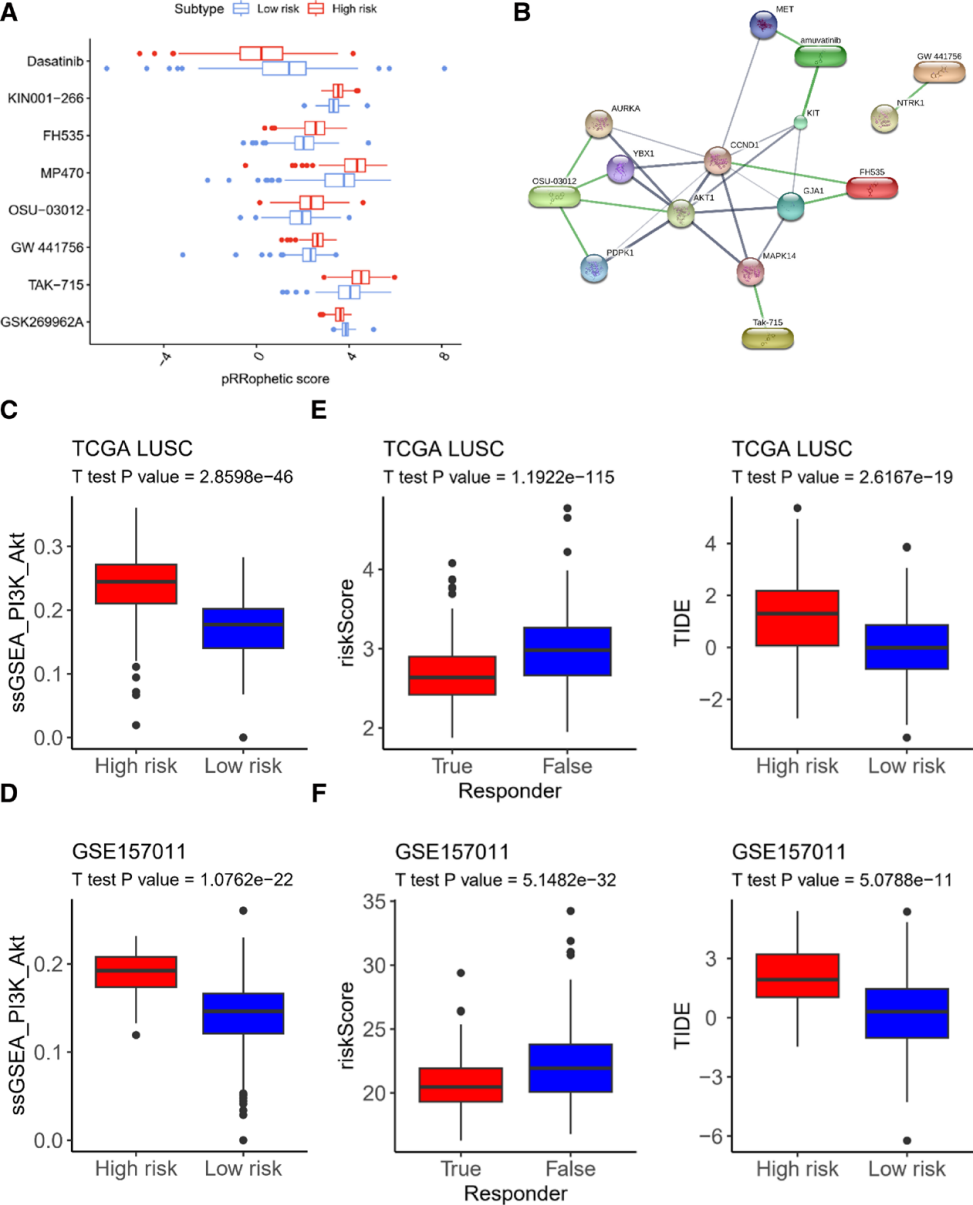
Prediction of effective Chemotherapy and Immunotherapy for TCGA-LUSC with a high or low-risk score. Box plots comparing pRRophetic scores of the overlapped chemotherapy drugs between high-and low–CAF risk groups in TCGA-LUSC (A). Schematic presentation of a protein–protein and protein-chemical association network for the high-risk sensitive overlapped chemotherapy based on the STITCH database (B). Box plots demonstrate significantly higher ssGSEA scores for PI3K-Akt signaling pathways in high-risk as compared to low-risk groups in TCGA-LUSC (C) and GSE157011 (D). TIDE immunotherapy prediction analyses in TCGA-LUSC (E) and GSE157011 (F). LUSC = lung squamous cell carcinoma, ssGSEA = single-sample gene set enrichment analysis, STITCH = search tool for interaction of chemicals, TCGA = the cancer genome atlas, TIDE = tumor immune dysfunction and exclusion.

We selected the top 30 chemotherapy compounds in terms of the false discovery rate between the high- and low-risk groups for the TCGA-LUSC and GSE157011 cohorts individually. Eight compounds overlapped; 6 of them were more sensitive in the high-risk group (TAK-715, GW 441756, OSU-03012, MP470, FH535, and KIN001-266) and 2 were more sensitive in the low-risk group (GSK269962A and dasatinib) (Fig. 6A, Figure S7A, Supplemental Digital Content, http://links.lww.com/MD/J734). Five high-risk sensitive compounds (TAK-715, GW 441756, OSU-03012, MP470, and FH535) had a close correlation with PI3K-Akt signaling (Fig. 6B–D, Figure S7B, Supplemental Digital Content, http://links.lww.com/MD/J734) according to STITCH database analysis (KIN001-266 was not found in the STITCH database).

According to TIDE immunotherapy prediction analysis, the low-risk group had a higher immunotherapy response rate than the high-risk group in both the TCGA-LUSC and GSE157011 cohorts (Fig. 6E and F).

## 4. Discussion

CAFs have been shown to play a crucial role in the development and progression of LUSC by modulating the TME^[11]^; regulating antitumor lymphocyte infiltrations; and contributing to LUSC progression, treatment resistance, and immunosuppression.^[12]^ We have herein described a novel CAF stratification for LUSC based on 8 genes obtained from a LASSO Cox regression model (DCBLD1, SLC24A3, ILK, SMAD7, SERPINE1, SNX9, PDGFA, and KLF10). Higher CAF abundance levels and stromal scores were correlated with poorer prognoses. We also found that the high-risk CAF groups were more sensitive to TAK-715, GW 441756, OSU-03012, MP470, FH535, and KIN001-266. Additionally, the STITCH database revealed PI3K-Akt signaling as potential target pathway for high-risk CAF-related LUSC. The data presented herein hold promise for CAF-driven prognostic stratification in patients with LUSC, which could potentially pave the way for more efficient and personalized molecular targeting of stromal CAFs in future clinical trials.

According to the results of the LASSO Cox analysis, 8 survival-related CAF candidate genes were identified, facilitating construction of a regression model in the TCGA-LUSC cohort. Two prognostic candidates in this gene signature are already well-known CAF-related genes. Ciszewski et al^[38]^ reported that ILK is a component of the ILK/MMP9/MRTF axis and is involved in the induction of mesenchymal transdifferentiation of human microvascular endothelial cells into CAF-like cells. This process is associated with changes in stress fiber organization, changes in cell morphology, increased levels of α-smooth muscle actin protein, and activation of the RhoA and Rac-1 pathways. Notably, several studies have shown that SERPINE1 is upregulated in CAFs; this gene encodes plasminogen activator inhibitor-1 (PAI-1), which is associated with a poor prognosis in certain types of cancer when present at high levels within tumor tissues. Additionally, PAI-1 produced by CAFs enhances cancer cell migration and invasion abilities by activating the Akt and Erk1/2 signaling pathways through interaction with the low-density lipoprotein receptor-related protein 1, which serves as a receptor for PAI-1.^[39,40]^

An in silico drug screening system predicted 6 potentially effective compounds for high-risk LUSC. According to the STITCH database, PI3K-Akt signaling seems to be the most important factor for establishment and maintenance of the CAF high-risk phenotype. This finding is supported by evidence of higher activity of PI3K-Akt signaling in high- than in low-risk LUSC.

The PI3K-Akt signaling pathway is a crucial factor in the regulation of CAF formation and infiltration within the TME. Multiple upstream factors, including exosomal miR-21 derived from hepatocellular carcinoma cells^[41]^ and soluble carcinoembryonic antigen released from colorectal cancer cells, sensitize the Akt pathway to activate CAFs and promote cancer progression.^[42]^

Akt signaling activation in CAFs exerts multifaceted effects on cancer progression. In lung cancer, Akt/mTORC1 signaling activation in CAFs increases MDM2 expression, promoting cell invasion.^[43]^ In oral squamous cell carcinoma, CAFs express high levels of integrin β2, which enhances their glycolytic activity by regulation of the PI3K/Akt/mTOR pathway. This metabolic reprogramming leads to an increase in lactate secretion by CAFs, and the lactate is then metabolized by cancer cells to produce nicotinamide adenine dinucleotide, ultimately supporting cancer cell proliferation.^[44]^ In breast cancer, C3a/C3aR signaling activates the PI3K/Akt pathway, leading to increased metastatic cytokine secretion and extracellular matrix generation by CAFs, ultimately facilitating tumor metastasis.^[45]^

More commonly, CAFs exert their effects on cancer progression by modulating the PI3K/Akt signaling activity in cancer cells. For instance, Lou et al^[46]^ revealed that connexin 43 (Cx43)-formed unidirectional gap junctional intercellular communication mediates metabolic coupling between CAFs and non-small cell lung cancer (NSCLC) cells, promoting NSCLC progression by activating the PI3K/Akt and MAPK/ERK pathways, thus underscoring the critical role of CAFs and gap junctional intercellular communication in lung cancer malignancy. Similarly, Li et al^[47]^ highlighted the significant role of CAF-secreted IL-22 in promoting NSCLC progression via the activation of PI3K-Akt-mTOR signaling. Additionally, Yang et al^[48]^ demonstrated that miR-210 carried by CAFs-derived exosomes plays a critical role in promoting EMT and NSCLC progression by targeting UPF1 and activating the PTEN/PI3K/AKT pathway, highlighting CAFs as important regulators in lung cancer metastasis. In other cancer types, such as gallbladder cancer, CAF-secreted thrombospondin 4 binds to integrin α2 on cancer cells, leading to Akt-dependent phosphorylation of heat shock factor 1 and maintenance of the cancer cell’s malignant phenotype (including proliferation, epithelial-to-mesenchymal transition, and stemness).^[49]^ In breast cancer, CAFs secrete collagen to activate the integrin β1/PI3K/Akt pathway, thus regulating microtubule-directed chemoresistance. Moreover, CAF-derived CXCL5 promotes programmed cell death-ligand-1 expression in cancer cells by activating the PI3K/Akt pathway, creating an immunosuppressive microenvironment.^[50]^

Our study revealed an intriguing association between CAFs and the forkhead box O (FoxO) signaling pathway in LUSC; this pathway is downstream of the PI3K-Akt signaling pathway. Zhang et al^[51]^ investigated the influence of the cross talk between CAFs and cancer cells on FoxO1 expression and activation. They discovered that tumor cells can stimulate CAF activation by upregulation of α-smooth muscle actin and production of transforming growth factor-β1 (TGFβ1). Notably, FoxO1 was found to enhance TGFβ1 promoter activity, leading to increased expression of TGFβ1 when phosphorylated at threonine 24. This in turn created a link between CAF-mediated resistance and a FoxO1–TGFβ1 loop.

Like other studies that integrate sequencing data from bulk tumor tissue, our research has several inherent limitations. For instance, a prospective analysis of a tremendously large cohort of patients with LUSC is needed to validate the LASSO Cox risk model. Furthermore, the effectiveness of targeting the PI3K-Akt signaling pathway for treating high-risk CAF-related LUSC requires further validation in appropriate preclinical models and future clinical trials. These findings emphasize the need for continued investigation to fully elucidate the potential clinical implications of these pathways in LUSC.

## 5. Conclusion

In summary, we identified 288 hub CAF-related candidate genes by WGCNA, and 34 potential prognostic CAF candidate genes were identified by univariate Cox regression in TCGA-LUSC cohort. Then we prioritized the top 8 CAF prognostic genes by LASSO Cox regression model, and these genes were used to identify low- and high-risk subgroups as well as unfavorable survival. In silico drug screening identified 6 effective compounds for high-risk CAF-related LUSC. Furthermore, STITCH database highlighted PI3K-Akt signaling as a potential target pathway for high-risk CAF-related LUSC. Overall, our findings provide a molecular classifier for high-risk CAF-related LUSC and suggest that treatment with PI3K-Akt signaling inhibitors could benefit these patients.

## Acknowledgements

We extend our heartfelt appreciation to the participants of the TCGA-LUSC and GSE157011 cohorts for providing their clinical information and mRNA expression data, which were crucial for our analysis. We also thank the researchers and staff involved in data collection and curation.

## Author contributions

**Conceptualization:** Kuailu Lin.

**Data curation:** Zuoliu Xie, Kuailu Lin.

**Formal analysis:** Xixi Lai, Gangze Fu, Zuoliu Xie.

**Investigation:** Xixi Lai, Haiyan Du, Saifeng Lin.

**Methodology:** Xixi Lai, Qiao Li, Kuailu Lin.

**Project administration:** Kuailu Lin.

**Writing – original draft:** Xixi Lai, Gangze Fu.

**Writing – review & editing:** Kuailu Lin.

## Supplementary Material


























