# Supplementary material for: Identification of a cancer-associated fibroblast classifier for predicting prognosis and therapeutic response in lung squamous cell carcinoma
Source: Medicine (Baltimore). 2023 Sep 22;102(38):e35005. doi: 10.1097/MD.0000000000035005 (PMC10519496; doi:10.1097/MD.0000000000035005)
Supplement: Supplementary file 2 [file medi-102-e35005-s002.pptx]

## Slide 1
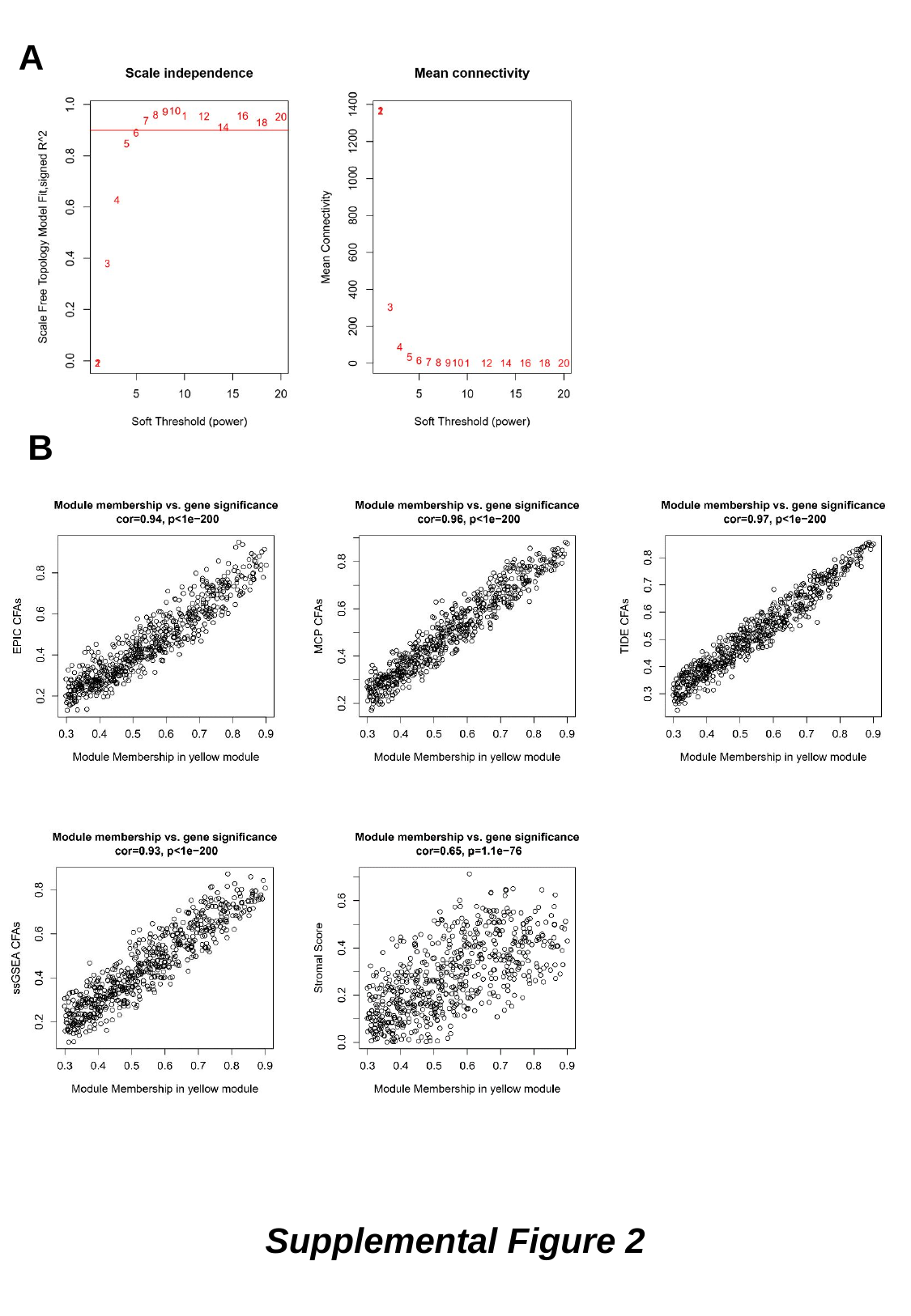

A
B
Supplemental Figure 2

## Slide 2
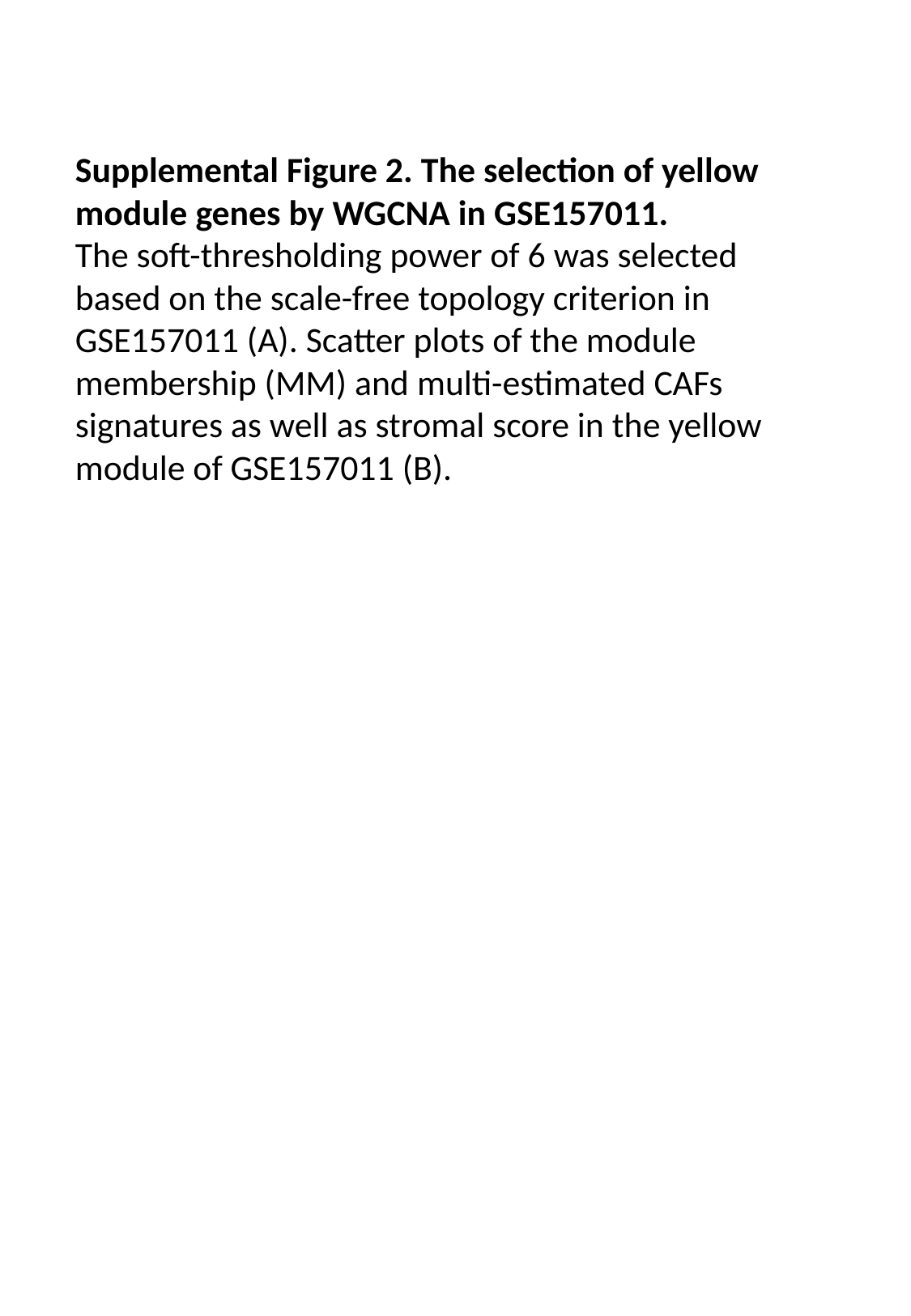

Supplemental Figure 2. The selection of yellow module genes by WGCNA in GSE157011.
The soft-thresholding power of 6 was selected based on the scale-free topology criterion in GSE157011 (A). Scatter plots of the module membership (MM) and multi-estimated CAFs signatures as well as stromal score in the yellow module of GSE157011 (B).
