# Supplementary material for: Identification of a cancer-associated fibroblast classifier for predicting prognosis and therapeutic response in lung squamous cell carcinoma
Source: Medicine (Baltimore). 2023 Sep 22;102(38):e35005. doi: 10.1097/MD.0000000000035005 (PMC10519496; doi:10.1097/MD.0000000000035005)
Supplement: Supplementary file 3 [file medi-102-e35005-s003.pptx]

## Slide 1
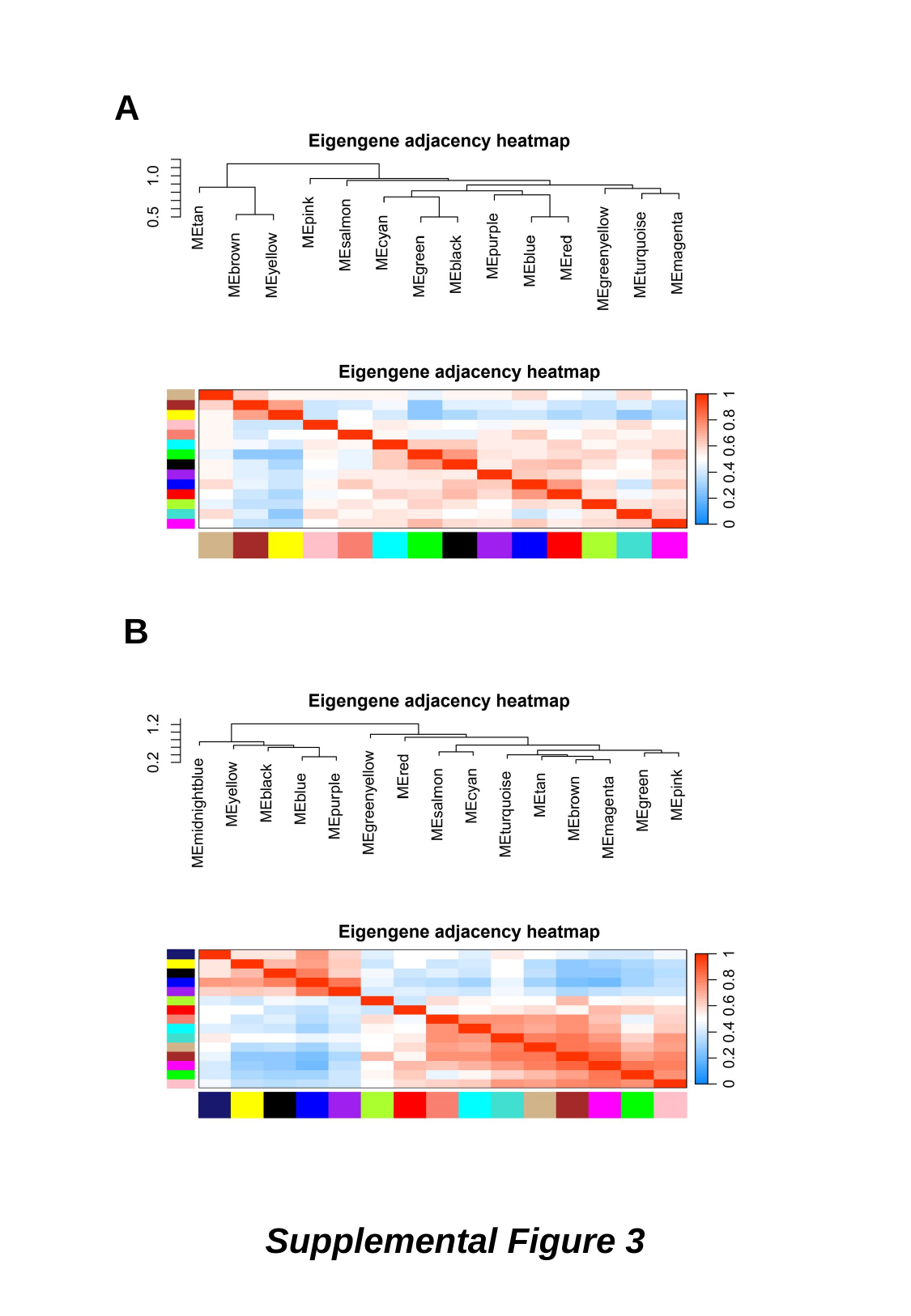

A
B
Supplemental Figure 3

## Slide 2
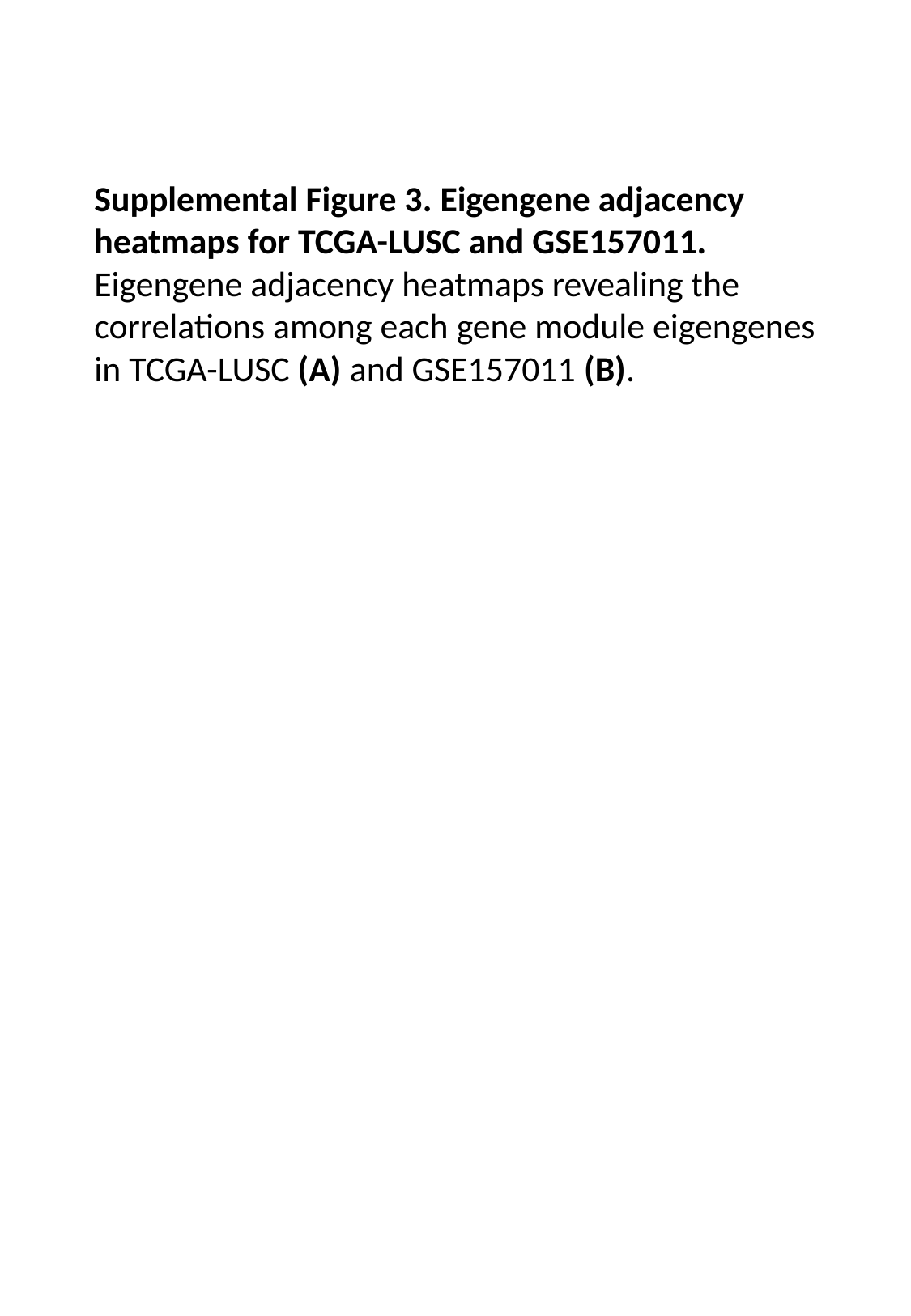

Supplemental Figure 3. Eigengene adjacency heatmaps for TCGA-LUSC and GSE157011.
Eigengene adjacency heatmaps revealing the correlations among each gene module eigengenes in TCGA-LUSC (A) and GSE157011 (B).
