# Supplementary material for: Identification of a cancer-associated fibroblast classifier for predicting prognosis and therapeutic response in lung squamous cell carcinoma
Source: Medicine (Baltimore). 2023 Sep 22;102(38):e35005. doi: 10.1097/MD.0000000000035005 (PMC10519496; doi:10.1097/MD.0000000000035005)
Supplement: Supplementary file 6 [file medi-102-e35005-s006.pptx]

## Slide 1
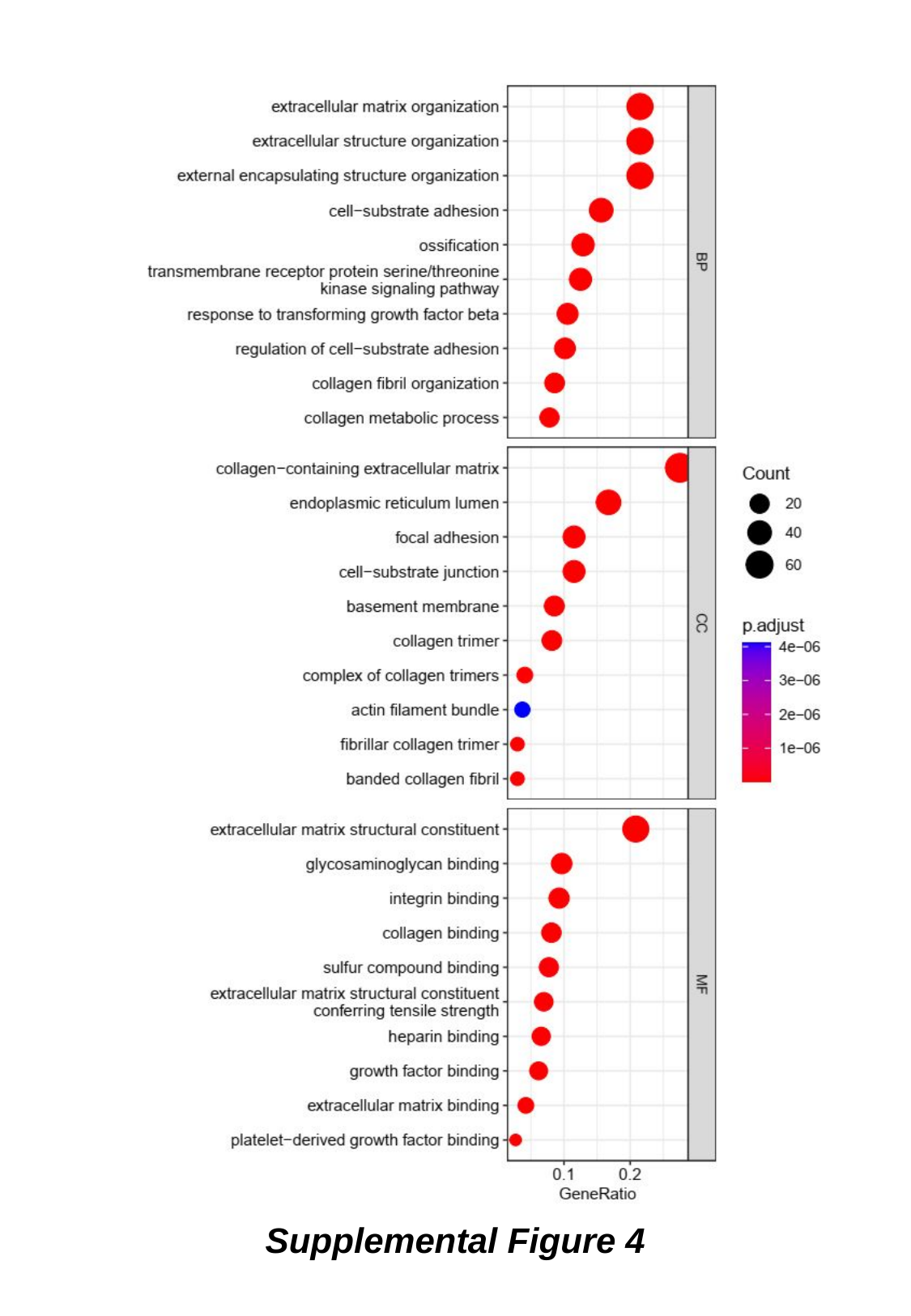

Supplemental Figure 4

## Slide 2
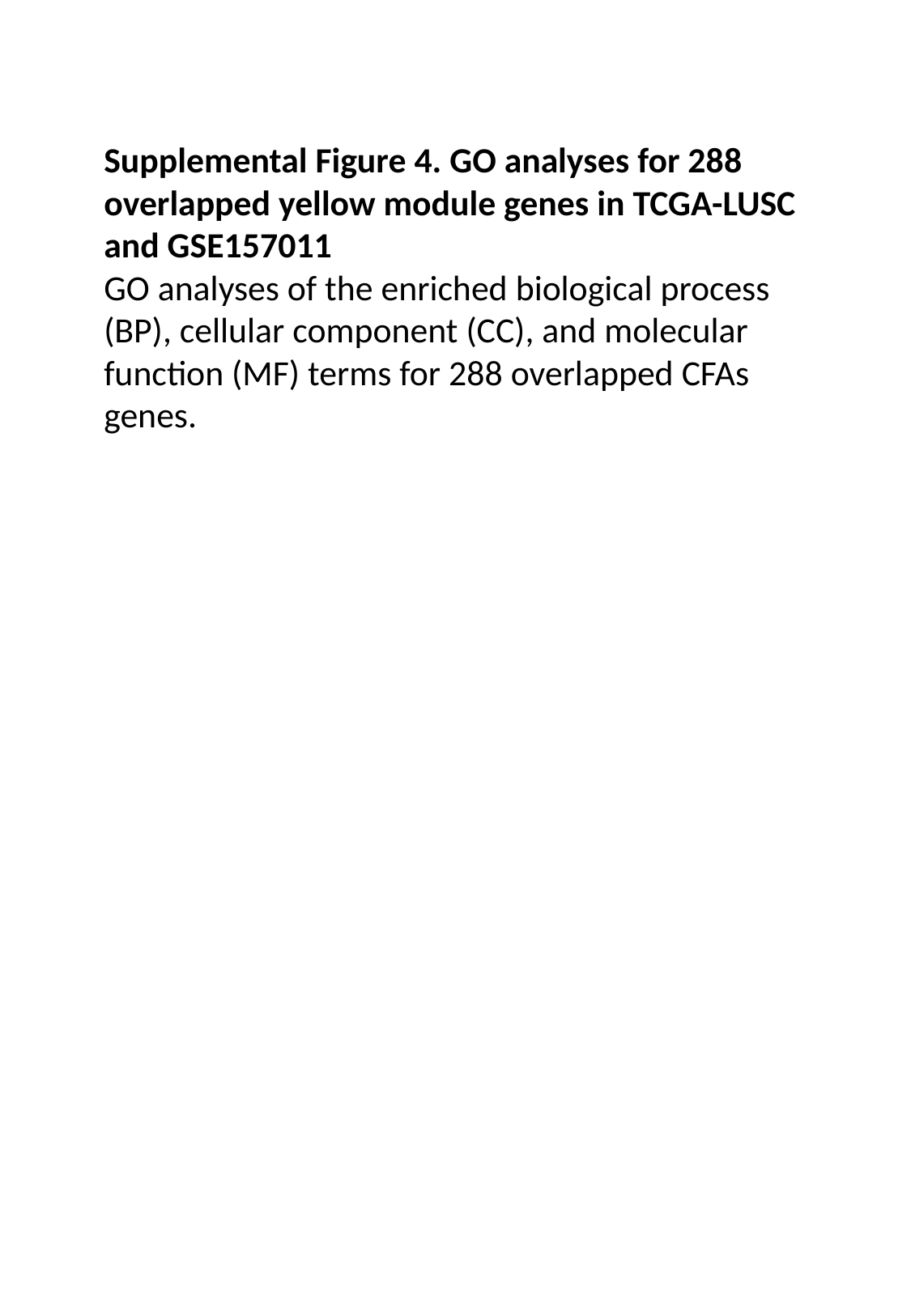

Supplemental Figure 4. GO analyses for 288 overlapped yellow module genes in TCGA-LUSC and GSE157011
GO analyses of the enriched biological process (BP), cellular component (CC), and molecular function (MF) terms for 288 overlapped CFAs genes.
