# Supplementary material for: Identification of a cancer-associated fibroblast classifier for predicting prognosis and therapeutic response in lung squamous cell carcinoma
Source: Medicine (Baltimore). 2023 Sep 22;102(38):e35005. doi: 10.1097/MD.0000000000035005 (PMC10519496; doi:10.1097/MD.0000000000035005)
Supplement: Supplementary file 9 [file medi-102-e35005-s009.pptx]

## Slide 1
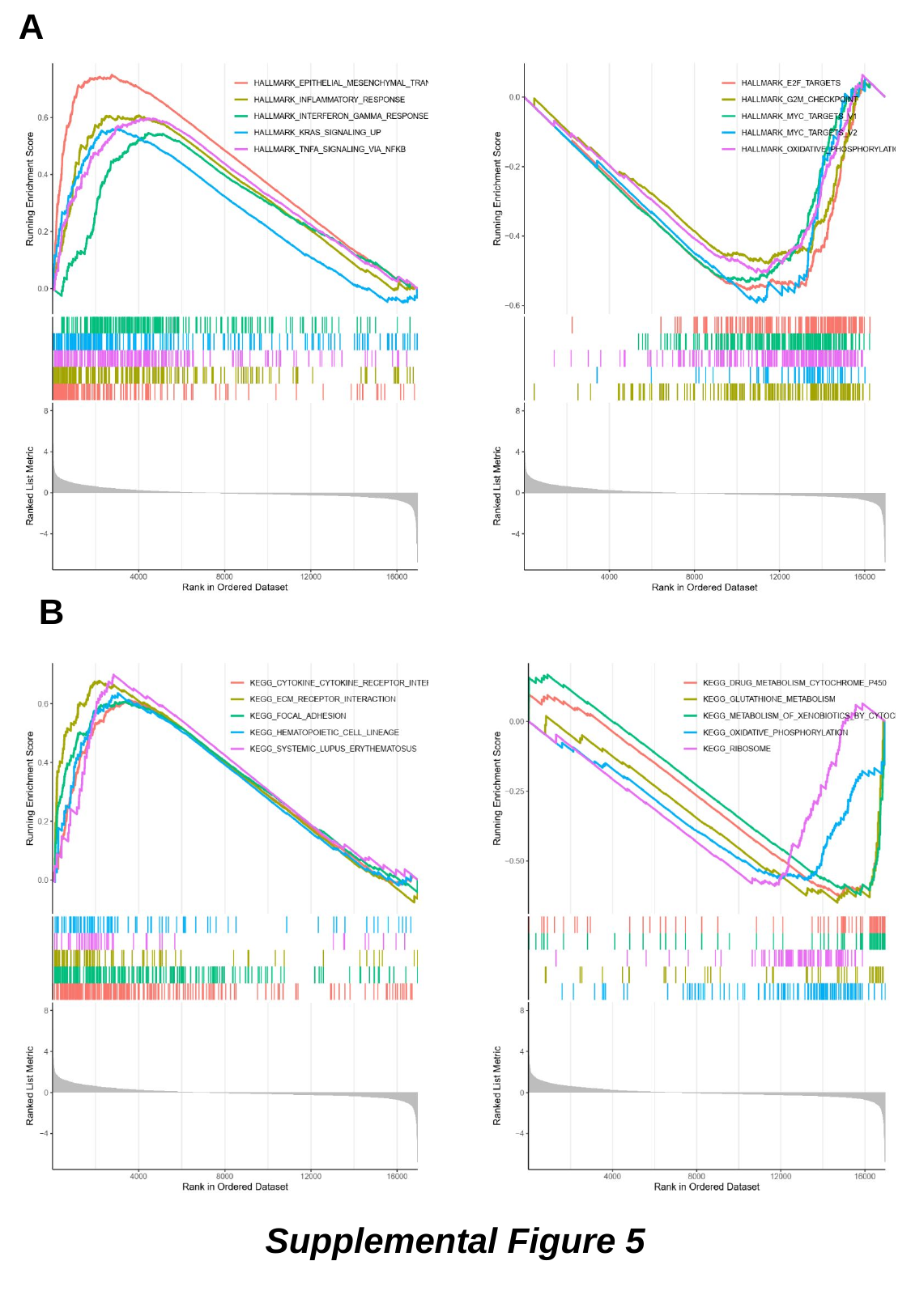

A
B
Supplemental Figure 5

## Slide 2
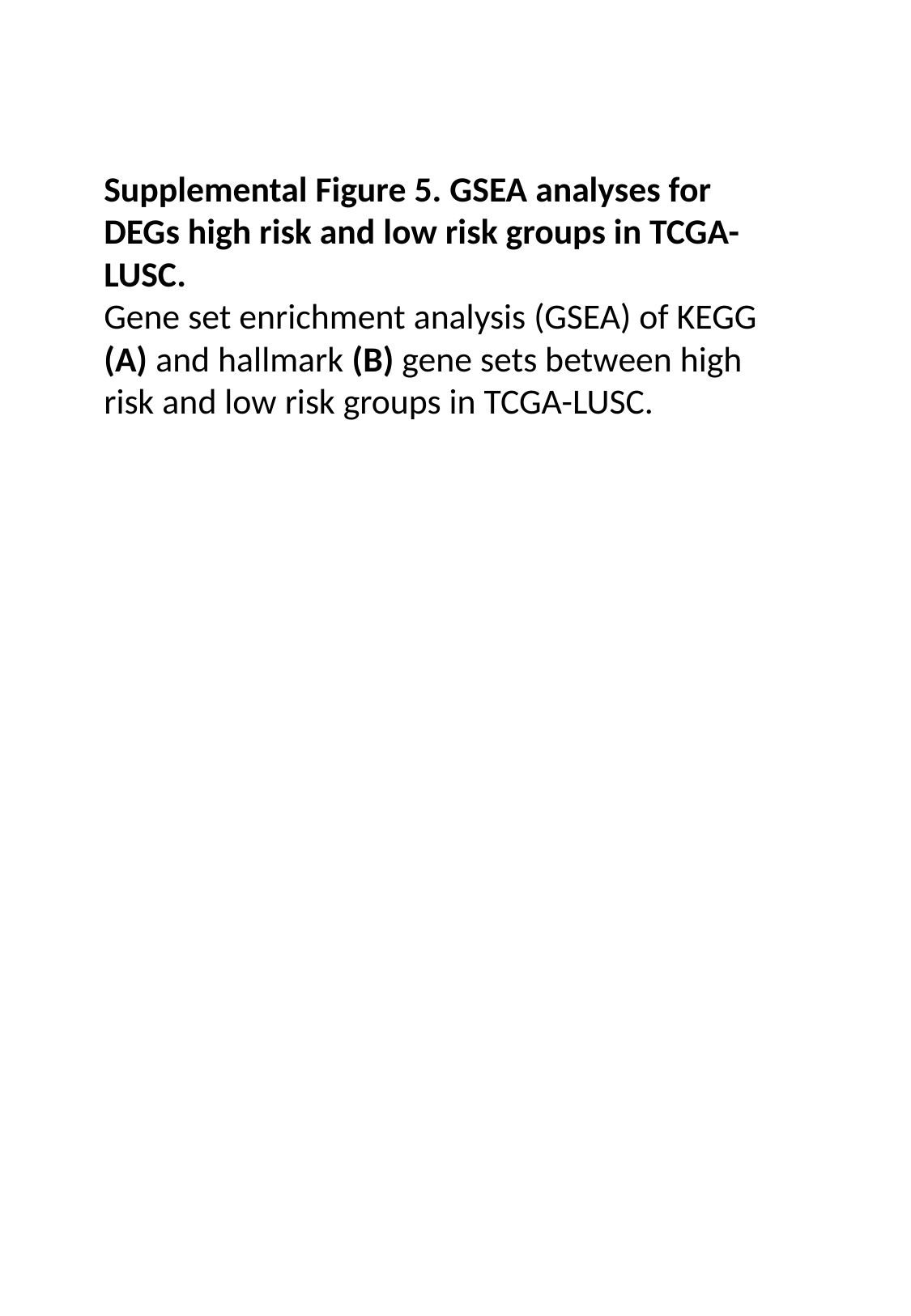

Supplemental Figure 5. GSEA analyses for DEGs high risk and low risk groups in TCGA-LUSC.
Gene set enrichment analysis (GSEA) of KEGG (A) and hallmark (B) gene sets between high risk and low risk groups in TCGA-LUSC.
