# Supplementary material for: Identification of a cancer-associated fibroblast classifier for predicting prognosis and therapeutic response in lung squamous cell carcinoma
Source: Medicine (Baltimore). 2023 Sep 22;102(38):e35005. doi: 10.1097/MD.0000000000035005 (PMC10519496; doi:10.1097/MD.0000000000035005)
Supplement: Supplementary file 12 [file medi-102-e35005-s012.pptx]

## Slide 1
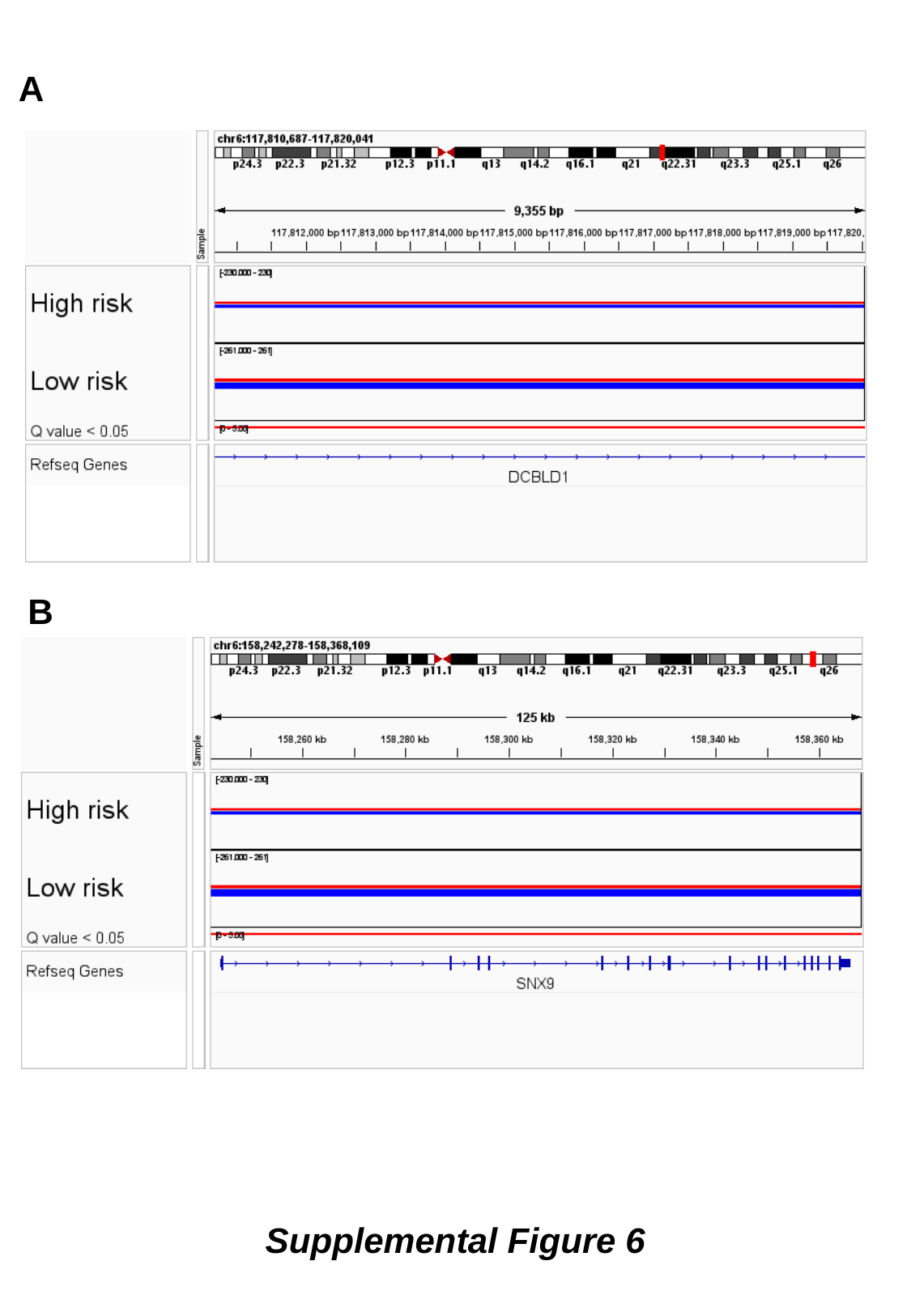

A
B
Supplemental Figure 6

## Slide 2
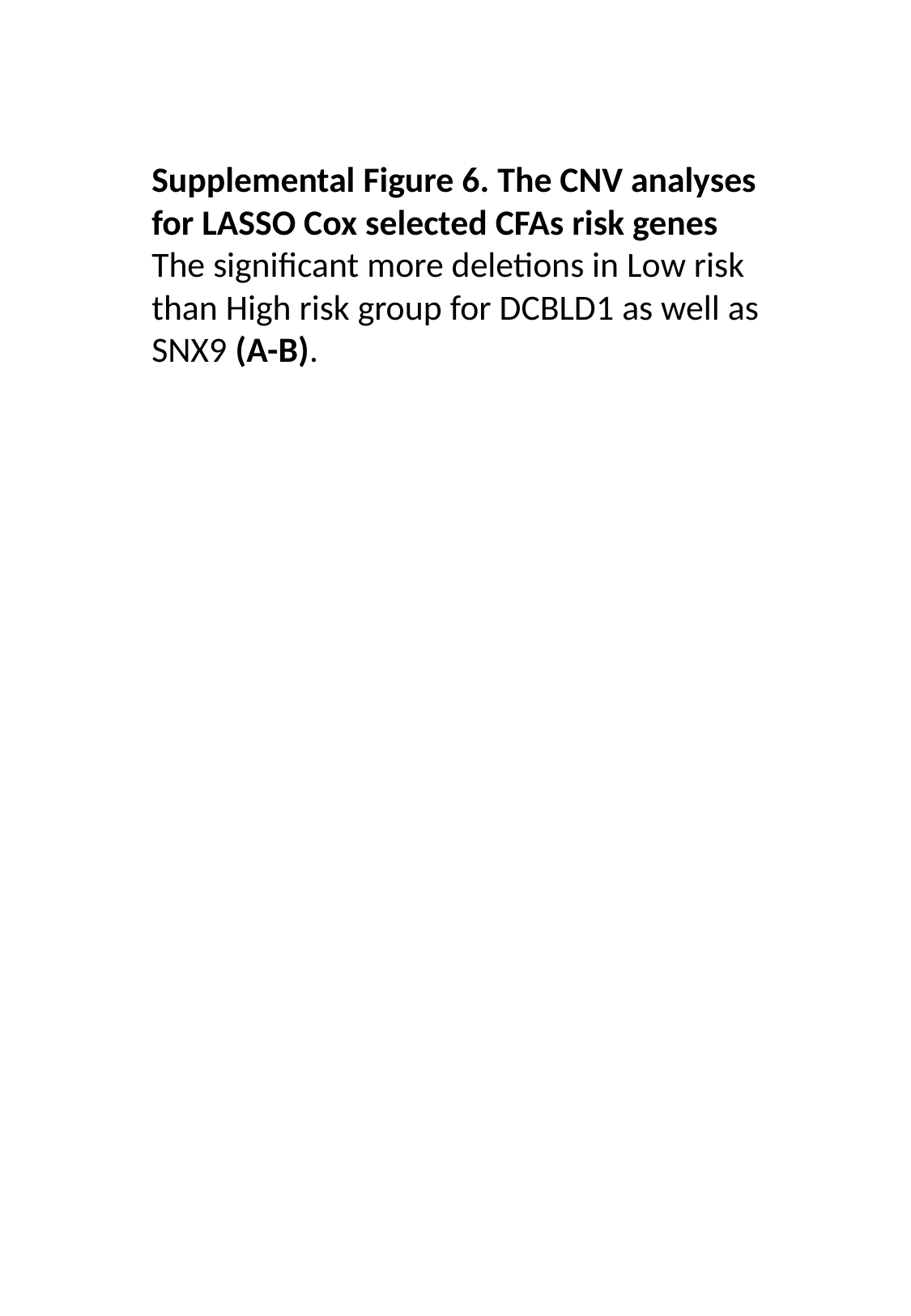

Supplemental Figure 6. The CNV analyses for LASSO Cox selected CFAs risk genes
The significant more deletions in Low risk than High risk group for DCBLD1 as well as SNX9 (A-B).
