# Supplementary material for: Identification of a cancer-associated fibroblast classifier for predicting prognosis and therapeutic response in lung squamous cell carcinoma
Source: Medicine (Baltimore). 2023 Sep 22;102(38):e35005. doi: 10.1097/MD.0000000000035005 (PMC10519496; doi:10.1097/MD.0000000000035005)
Supplement: Supplementary file 13 [file medi-102-e35005-s013.pptx]

## Slide 1
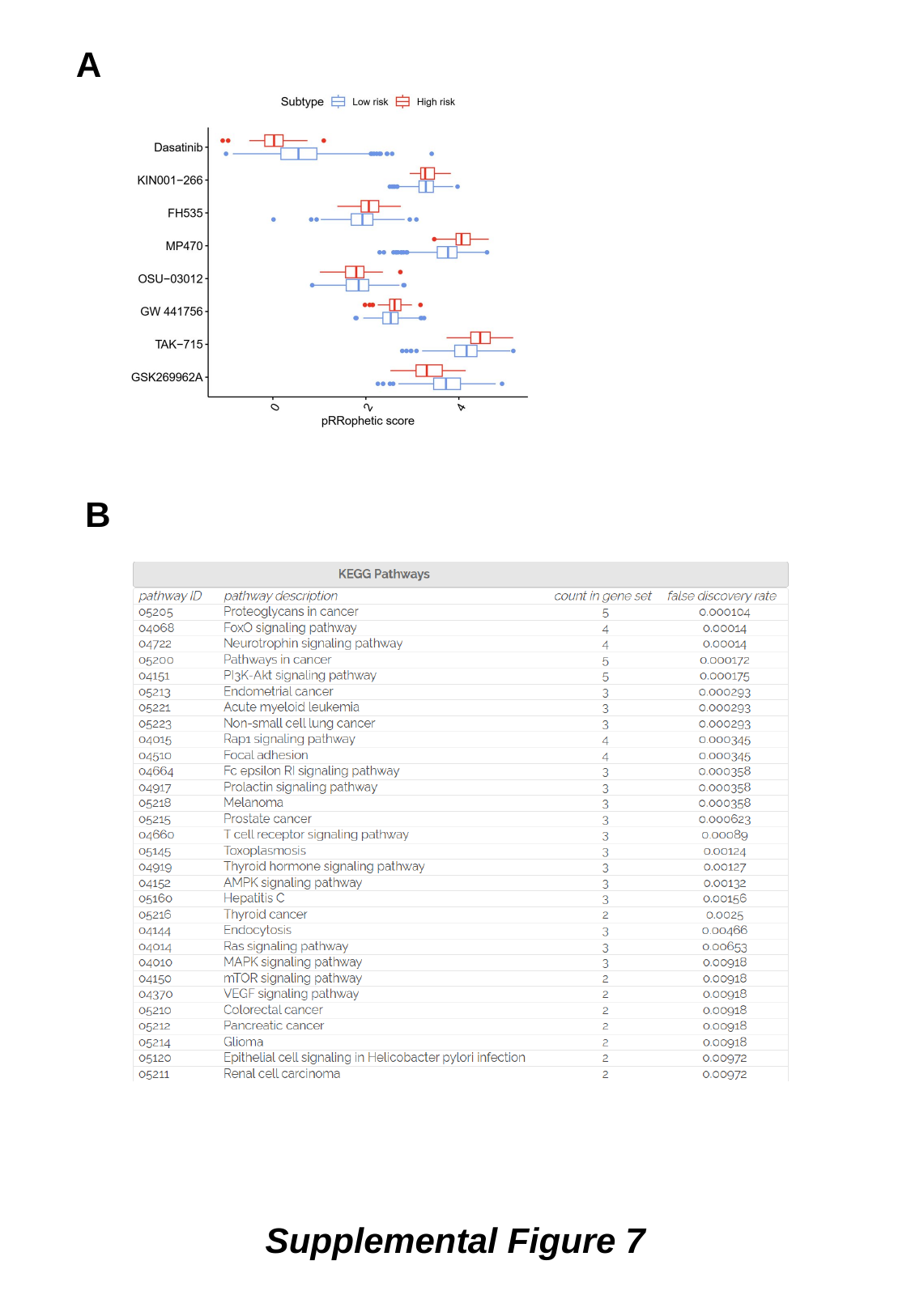

A
B
Supplemental Figure 7

## Slide 2
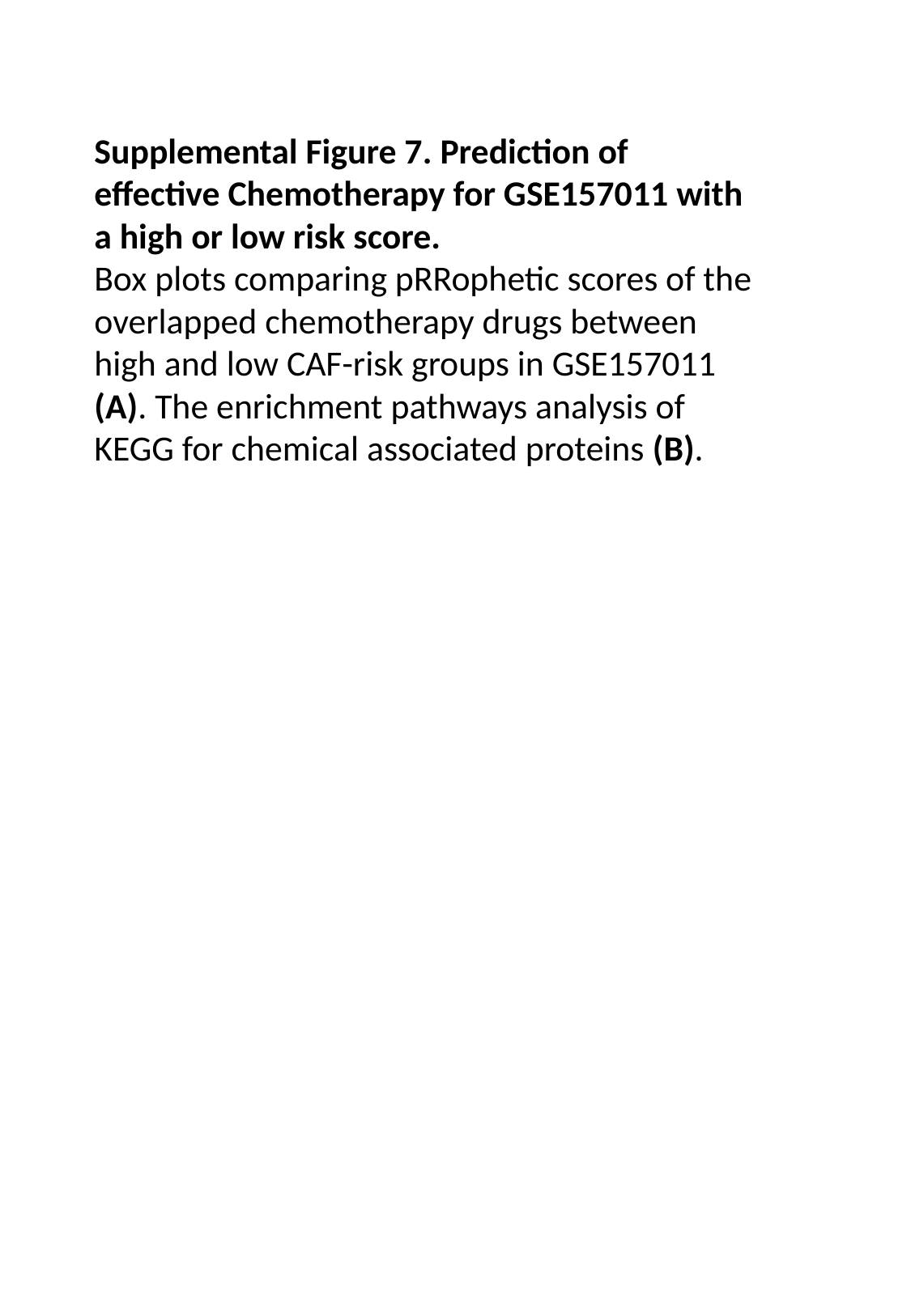

Supplemental Figure 7. Prediction of effective Chemotherapy for GSE157011 with a high or low risk score.
Box plots comparing pRRophetic scores of the overlapped chemotherapy drugs between high and low CAF-risk groups in GSE157011 (A). The enrichment pathways analysis of KEGG for chemical associated proteins (B).
